# Socioeconomic Status, Modifiable Factors, and Risk of Microvascular Complications in Individuals With Type 2 Diabetes: A Cohort Study From the UK Biobank

**DOI:** 10.1111/1753-0407.70079

**Published:** 2025-04-02

**Authors:** Ying Li, Qiqi You, Menglin Fan, Lingqi Wei, Jingjing Zeng, Bo Chen, Jie Wang, Shaoyong Xu

**Affiliations:** ^1^ College of Medicine Wuhan University of Science & Technology Wuhan Hubei China; ^2^ Department of Endocrinology, Xiangyang Central Hospital Affiliated Hospital of Hubei University of Arts and Science Xiangyang Hubei China; ^3^ Center for Clinical Evidence‐Based and Translational Medicine, Xiangyang Central Hospital Affiliated Hospital of Hubei University of Arts and Science Xiangyang Hubei China; ^4^ Wuhan Institute of Physics and Mathematics, Innovation Academy for Precision Measurement Science and Technology Chinese Academy of Sciences Wuhan Hubei China

**Keywords:** cohort, mediation analysis, microvascular complication, socioeconomic status, type 2 diabetes

## Abstract

**Background:**

To investigate whether lower socioeconomic status (SES) was associated with an increased risk of diabetic microvascular complications and to analyze the potential mediating role of several modifiable factors.

**Methods:**

The study included 11 309 patients with type 2 diabetes at baseline from the UK Biobank cohort. SES was grouped based on income, education, and employment status by using latent class analysis. Microvascular complications of diabetes were identified through electronic health records. Cox regression models were used to estimate hazard ratios (HRs) and 95% confidence intervals (CIs) for microvascular complications across SES groups. Mediation analysis was applied to explore potential mediators in these associations.

**Results:**

During a median follow‐up of 12.2 years, 262, 764, and 1017 participants in the high, medium, and low SES groups were diagnosed with microvascular complications. Compared to participants with high SES, those with low SES had a HR of 1.75 (95% CI: 1.53, 2.01) for total microvascular complications, a HR of 2.11 (95% CI: 1.74, 2.55) for nephropathy, a HR of 1.40 (95% CI: 1.14, 1.72) for retinopathy, and a HR of 1.79 (95% CI: 1.32, 2.43) for neuropathy. Mediation analysis indicated that alcohol consumption, body mass index, triglycerides, high density lipoprotein cholesterol, and glycated hemoglobin mediated the association between SES and microvascular complications, with mediation percentages of 1.3%, 12.2%, 4.4%, 10.9%, and 10.8%, respectively.

**Conclusions:**

Lower SES may be associated with a higher risk of diabetic microvascular complications, and obesity‐related indicators and glycated hemoglobin may play important mediating roles in the association.

## Graphical abstract

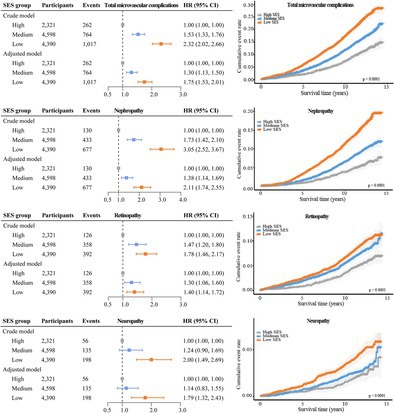

**Summary**

- Socioeconomic status (SES) is a comprehensive measure of individual economic and social standing, and socioeconomic inequalities may play a significant role in the development of chronic metabolic diseases.
- Lower SES was associated with a higher risk of diabetic microvascular complications. Obesity‐related indicators and glycated hemoglobin may play important mediating roles.
- SES is relatively difficult to change, and efforts are suggested to be made through other approaches to prevent or reduce the occurrence of diabetic microvascular complications.

## 1 Introduction

Diabetes is one of the most prevalent chronic diseases and affects over 500 million adults worldwide, which is primarily characterized by high blood glucose. Long‐term exposure to high blood glucose levels adversely affects microvasculature and may lead to microvascular complications [1]. It is estimated that diabetic nephropathy and retinopathy affected approximately 25% of patients with type 2 diabetes (T2D) and diabetic neuropathy occurred in almost 50% of the diabetic population [2]. Diabetes and its microvascular complications have a significant impact on the life quality and overall life expectancy of diabetic patients, as well as impose a substantial burden on the healthcare system [3].

Socioeconomic status (SES) is a comprehensive measure of individual economic and social standing [4], and socioeconomic inequalities may play a significant role in behavioral risk factors or the development of chronic metabolic diseases [5, 6, 7]. Some studies have found an effect of SES on diabetic microvascular complications [8, 9, 10, 11, 12, 13]. However, to our knowledge, they were primarily cross‐sectional designs and tended to focus on diabetic nephropathy and retinopathy, with less focus on diabetic neuropathy [14]. In addition, since SES is relatively difficult to change, much attention has been paid to modifiable factors (e.g., lifestyle risks) as possible mediators of the association between SES and health outcomes [15]. For example, a study by Zhang et al. [16] found that low SES was associated with higher all‐cause mortality and cardiovascular disease risk, with 4.0% and 3.7% of the association mediated by lifestyle factors, respectively. We are particularly interested in whether modifiable factors influence the association between SES and diabetic microvascular complications, but relevant studies are lacking.

Recently, the latent class analysis has been popularly used to assess SES based on education level, income, and occupation [4, 16, 17], which could provide more comprehensive evidence. Our study aimed to investigate whether lower SES defined using this approach was associated with an increased risk of diabetic microvascular complications and to analyze the potential mediating role of some modifiable factors using data from the UK Biobank.

## 2 Methods

### 2.1 Data Source and Study Population

UK Biobank is an ongoing, open, dynamic, and large‐scale cohort conducted in the United Kingdom. It comprises over 500 000 participants aged between 40 and 69 recruited from 22 locations across England, Scotland, and Wales between 2006 and 2010 [18]. Data collection primarily involves touchscreen questionnaires, physical measurements, biological samples, and linkage with public sectors such as National Health Service England, Information and Statistics Division Scotland, and so forth. Further information regarding UK Biobank can be found at https://www.ukbiobank.ac.uk.

We identified 11 563 T2D patients with data for primary variables at baseline. The diagnosis of diabetes was ascertained by the International Classification of Diseases, 10th edition (ICD‐10) codes, as well as blood glucose and glycated hemoglobin assessments [19]. Patients were excluded if they had already developed microvascular complications of diabetes at baseline (*n* = 254). Ultimately, 11 309 T2D patients were included in the analysis (Figure S1).

### 2.2 Ascertainment of SES

We assessed SES based on three variables: average total household income before tax, education, and employment status. Information on them was obtained through touch‐screen questionnaires. Regarding the average total household income before tax, participants had the following options: (1) < £18 000; (2) £18 000–£30 999; (3) £31 000–£51 999; (4) £52 000–£100 000; (5) > £100 000; (6) Do not know; (7) Prefer not to answer. We excluded participants who chose the last two options [16]. Options of education were as follows: (1) College or University degree; (2) A levels/AS levels or equivalent; (3) O levels/GCSEs or equivalent; (4) CSEs or equivalent; (5) NVQ or HND or HNC or equivalent; (6) Other professional qualifications; (7) None of the above (equivalent to less than high school diploma); (8) Prefer not to answer. We excluded individuals who chose the last option [16]. For the assessment of employment status, employment was defined as meeting any of the following criteria: (1) In paid employment or self‐employed; (2) Retired; (3) Doing unpaid or voluntary work; (4) Full or part‐time student, with those selecting other options defined as unemployed [16] (Table S1).

Based on the aforementioned three variables, individual‐level SES was estimated by using the latent class analysis, which is a probabilistic modeling algorithm that allows clustering of data and statistical inference [20]. Three categories representing high, medium, and low SES were identified based on item response probabilities (Tables S2 and S3). Additionally, the Townsend deprivation index was utilized to represent area‐level SES, which was incorporated into our sensitivity analysis. The Townsend deprivation index comprised composite scores of four key variables: unemployment, overcrowded households, lack of car ownership, and lack of home ownership, with higher scores indicating higher levels of poverty [4].

### 2.3 Ascertainment of Modifiable Factors and Other Covariates

We primarily assessed four lifestyle factors, including smoking status, alcohol consumption status, diet, and physical activity. These data were obtained through touchscreen questionnaires in the UK Biobank. No current smoking was defined as meeting one of the following criteria: (1) Never smoking; (2) Former smoker who had quit for more than 30 years [4]. No alcohol consumption was defined as never alcohol drinking [4]. Diet was evaluated based on daily or weekly intake of fruits, vegetables, fish, meat, and grains. A healthy diet was defined as meeting at least four of the following: (1) Daily intake of ≥ 4 servings of fruits; (2) Daily intake of ≥ 4 servings of vegetables; (3) Weekly intake of fish ≥ 2 times; (4) Weekly intake of processed meat ≤ 1 time; (5) Weekly intake of unprocessed red meat ≤ 1.5 times; (6) Daily intake of ≥ 3 servings of whole grains [4]. Physical activity was recorded as whether regular physical exercise was performed. Regular physical activity was defined as meeting at least one of the following: (1) Moderate exercise for no less than 150 min per week; (2) Vigorous exercise for no less than 75 min per week; (3) (moderate exercise time + 2 × vigorous exercise time) ≥ 150 min per week [21] (Table S1).

Height and weight were measured by trained nurses at the initial assessment visit. Height was measured using a Seca 202 device for standing height, while weight was acquired through various methods. Body mass index was calculated by dividing weight (in kilograms) by the square of height (in meters). Blood pressure was obtained as the average of two automatic readings using an Omron device. Metabolic markers were measured using blood samples collected at recruitment. Blood lipids, blood glucose, and creatinine were measured using a Beckman Coulter AU5800 device, and glycated hemoglobin was measured using a Bio‐Rad VARIANT II Turbo device. Glomerular filtration rate was calculated using the 2021 CKD‐EPI creatinine [22].

Other covariates including age, sex, ethnicity, insulin use, medication for cholesterol, and medication for blood pressure were obtained through touchscreen questionnaires and self‐reports. Ethnicity was categorized into five groups: White, Mixed, Asian, Black, and Other. Diabetes duration was calculated by subtracting the age at diabetes diagnosis from the age at enrollment.

### 2.4 Ascertainment of Outcome

The primary endpoint was the occurrence of diabetic microvascular complications, including diabetic nephropathy, diabetic neuropathy, and diabetic retinopathy. They were defined by the ICD‐10 codes, which were obtained by linkages with hospital inpatient admissions and death registries. According to previous literature [23], the ICD‐10 codes for diabetic nephropathy were: E11.2, E14.2, N08.3, N18.0, N18.1, N18.2, N18.3, N18.4, N18.5, N18.8, N18.9; the ICD‐10 codes for diabetic retinopathy were: E11.3, E14.3, H28.0, H36.0; and the ICD‐10 codes for diabetic neuropathy were: E11.4, E14.4, G59.0, G62.9, G63.2, G99.0.

### 2.5 Statistical Analysis

All statistical analyses were conducted using Stata 17.0 (Stata Corp, College Station, TX, USA) and RStudio (version 2023.09.1) software. Continuous variables were described using mean ± standard deviation, and categorical variables were described using number (percentage). Between‐group differences were compared by one‐way analysis of variance or Chi‐square test. A *p* value < 0.05 was considered statistically significant.

Cox proportional hazards regression models were used to estimate the hazard ratios (HRs) and corresponding 95% confidence intervals (CIs) of total and individual microvascular complications in participants from different SES groups. Participants were followed up from the recruitment date until the occurrence of outcome events, death, or the censoring date (September 30, 2021), whichever came first. Follow‐up time was measured in years. In the base model (Model 1), adjustments were made for age, sex, ethnicity, blood glucose, diabetes duration, insulin use, medication for cholesterol, and medication for blood pressure. To analyze the impact of some modifiable factors, we further made the following adjustments. First, lifestyle factors, body mass index, and metabolic markers were separately entered on top of Model 1 (Model 1+). Subsequently, all lifestyle factors were entered (Model 2), followed by all lifestyle factors plus body mass index (Model 3). Finally, all modifiable factors were simultaneously included in a comprehensive multivariable model (Model 4). The percentage difference of *β*_SES_ after including modifiable factors in the base model was calculated to determine the contribution of each modifiable factor in explaining the association between SES and diabetic microvascular complications. The formula for calculation was: 100 × (*β*_SES+modifiable factor(s)_ − *β*_SES_)/(*β*_SES_). CIs for the difference percentage were obtained using the bootstrap method with 1000 resamples [24].

We further examined whether the aforementioned modifiable factors were mediators between SES and diabetic microvascular complications. This process was conducted using the mediation package in R software, which estimated the direct effects, indirect effects, and total effects of SES on diabetic microvascular complications, as well as the percentage of mediation by the modifiable factors [25].

Subgroup analyses were performed concerning age, sex, and diabetes duration. We also examined the stability of the results by excluding participants whose outcomes occurred within 2 years and utilizing the Townsend deprivation index to reflect area‐level SES. Considering that the glomerular filtration rate may have an impact on diabetic nephropathy, we further adjusted the glomerular filtration rate in the sensitivity analysis.

## 3 Results

### 3.1 Baseline Characteristics of Study Population

The mean age of participants was 59.2 years, with males comprising 61.8% of the sample. The majority of the participants were White, accounting for 90.1%. There were 2321 (20.5%), 4598 (40.7%), and 4390 (38.8%) participants in the high, middle, and low SES group, respectively. Compared to participants with low SES, a higher proportion of males, White people, no current smokers, and adhering to a healthy diet was observed among those with high SES (*p* < 0.05). They also had a shorter duration of diabetes, lower body mass index and systolic blood pressure, lower levels of triglycerides and glycated hemoglobin, and higher levels of high‐density lipoprotein cholesterol and glomerular filtration rate (*p* < 0.05). No alcohol consumption, regular physical activity, insulin use, and medication for cholesterol and blood pressure were more prevalent among participants in the low SES group than in the high SES group (*p* < 0.05). No significant differences were found in blood glucose, cholesterol, and low‐density lipoprotein cholesterol among SES groups (*p* > 0.05). (Table 1).

**TABLE 1:** Baseline characteristics of study population.

Baseline characteristics^a^	Total population (*n* = 11 309)	High SES (*n* = 2321)	Medium SES (*n* = 4598)	Low SES (*n* = 4390)	*p*
Age (year)	59.2 ± 7.2	56.7 ± 7.4	59.0 ± 7.2	60.9 ± 6.7	< 0.001
Male, *n* (%)	6986 (61.8)	1506 (64.9)	2962 (64.4)	2518 (57.4)	< 0.001
White, *n* (%)	10 184 (90.1)	2121 (91.4)	4173 (90.8)	3890 (88.6)	< 0.001
Average total household income before tax (£)					< 0.001
Greater than ₤100 000	347 (3.1)	332 (14.3)	0 (0.0)	15 (0.3)	
₤52 000–100 000	1546 (13.7)	1034 (44.6)	506 (11.0)	6 (0.1)	
₤31 000–51 999	2543 (22.5)	955 (41.2)	1543 (33.6)	45 (1.0)	
₤18 000–30 999	3270 (28.9)	0 (0.0)	2549 (55.4)	721 (16.4)	
Less than ₤18 000	3603 (31.9)	0 (0.0)	0 (0.0)	3603 (82.1)	
Education level					< 0.001
College or university degree	3319 (29.4)	1998 (86.1)	779 (16.9)	542 (12.4)	
A/AS levels or equivalent	1184 (10.5)	260 (11.2)	647 (14.1)	277 (6.3)	
O/GCSEs level or equivalent	2324 (20.6)	28 (1.2)	1500 (32.6)	796 (18.1)	
CSEs or equivalent	541 (4.8)	2 (0.1)	319 (6.9)	220 (5.0)	
NVQ/HND/HNC or equivalent	997 (8.8)	15 (0.7)	625 (13.6)	357 (8.1)	
Other professional qualifications	696 (6.2)	18 (0.8)	460 (10.0)	218 (5.0)	
None of the above	2248 (19.9)	0 (0.0)	268 (5.8)	1980 (45.1)	
Employment status					< 0.001
Employed	10 330 (91.3)	2233 (96.2)	4598 (100.0)	3499 (79.7)	
Unemployed	979 (8.7)	88 (3.8)	0 (0.0)	891 (20.3)	
Diabetes duration, *n* (%)					< 0.001
< 5 years	9562 (84.6)	2054 (88.5)	3933 (85.5)	3575 (81.4)	
≥ 5 years	1747 (15.5)	267 (11.5)	665 (14.5)	815 (18.6)	
Insulin use, *n* (%)	1171 (10.4)	226 (9.7)	447 (9.7)	498 (11.3)	0.023
Medication for cholesterol, *n* (%)	6622 (58.6)	1182 (50.9)	2664 (57.9)	2776 (63.2)	< 0.001
Medication for blood pressure, *n* (%)	5878 (52.0)	982 (42.3)	2401 (52.2)	2495 (56.8)	< 0.001
No current smoking, *n* (%)	6640 (58.7)	1568 (67.6)	2718 (59.1)	2354 (53.6)	< 0.001
No alcohol consumption, *n* (%)	761 (6.7)	95 (4.1)	234 (5.1)	432 (9.8)	< 0.001
Healthy diet, *n* (%)	5588 (49.4)	1212 (52.2)	2260 (49.2)	2116 (48.2)	0.007
Regular physical activity, *n* (%)	8584 (75.9)	1720 (74.1)	3541 (77.0)	3323 (75.7)	0.026
Body mass index (kg/m^2^)	30.6 ± 5.6	29.6 ± 5.5	30.5 ± 5.5	31.2 ± 5.7	0.003
Systolic blood pressure (mmHg)	141.8 ± 17.7	139.9 ± 17.2	142.2 ± 17.6	142.4 ± 18.1	0.014
Blood glucose (mmol/L)	8.0 ± 3.0	8.2 ± 3.0	8.0 ± 3.1	7.8 ± 3.1	0.185
Glycated hemoglobin (mmol/mol)	51.3 ± 14.8	49.5 ± 15.0	51.5 ± 14.4	52.2 ± 14.9	0.014
Cholesterol (mmol/L)	4.9 ± 1.2	5.0 ± 1.2	4.9 ± 1.2	4.8 ± 1.2	0.701
Triglycerides (mmol/L)	2.2 ± 1.3	2.1 ± 1.3	2.3 ± 1.3	2.3 ± 1.3	0.002
High density lipoprotein cholesterol (mmol/l)	1.2 ± 0.3	1.3 ± 0.4	1.2 ± 0.3	1.2 ± 0.3	< 0.001
Low density lipoprotein cholesterol (mmol/L)	3.0 ± 0.9	3.1 ± 0.9	3.0 ± 0.9	2.9 ± 0.9	0.860
Glomerular filtration rate (mL/min/1.73 m^2^)	93.9 ± 14.1	96.8 ± 12.8	94.3 ± 13.8	92.0 ± 14.7	< 0.001

*Note:* SES is derived from the latent class analysis using information on household income, education, and employment status.

Abbreviation: SES, socioeconomic status.

^a^ Continuous variables were expressed as mean ± standard deviation, and categorical variables were expressed as number (percentage).

### 3.2 Association Between SES and Diabetic Microvascular Complications

**FIGURE 1:**
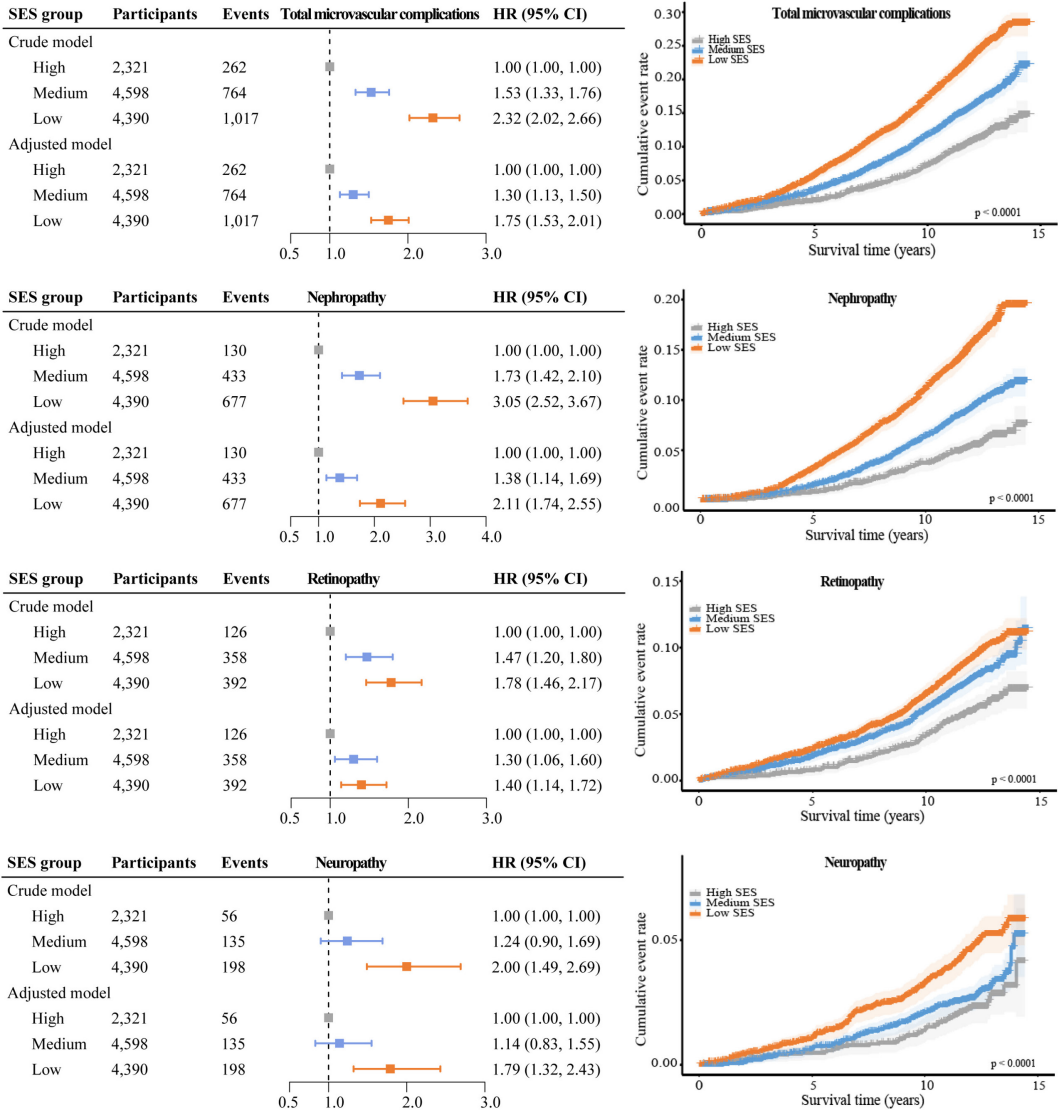
Association between SES group and diabetic microvascular complications. Crude model: Unadjusted; Adjusted model: Adjusted for age, sex, ethnicity, blood glucose, diabetes duration, insulin use, medication for cholesterol, and medication for blood pressure. CI, confidence interval; HR, hazard ratio; SES, socioeconomic status.

During a median follow‐up of 12.2 years, 2043 cases of diabetic microvascular complications were recorded, including 1240 cases of diabetic nephropathy, 876 cases of diabetic retinopathy, and 389 cases of diabetic neuropathy. Compared to individuals with high SES, those with medium and low SES had a HR of 1.30 (95% CI: 1.13, 1.50) and a HR of 1.75 (95% CI: 1.53, 2.01), respectively, for total microvascular complications. Specifically, participants with low SES had a 111% increase in risk for diabetic nephropathy (HR: 2.11, 95% CI: 1.74, 2.55), a 40% increase in risk for diabetic retinopathy (HR: 1.40, 95% CI: 1.14, 1.72), and a 79% increase in risk for diabetic neuropathy (HR: 1.79, 95% CI: 1.32, 2.43) compared to those with high SES (Figure 1).

### 3.3 Association Between SES, Modifiable Factors, and Diabetic Microvascular Complications

**FIGURE 2:**
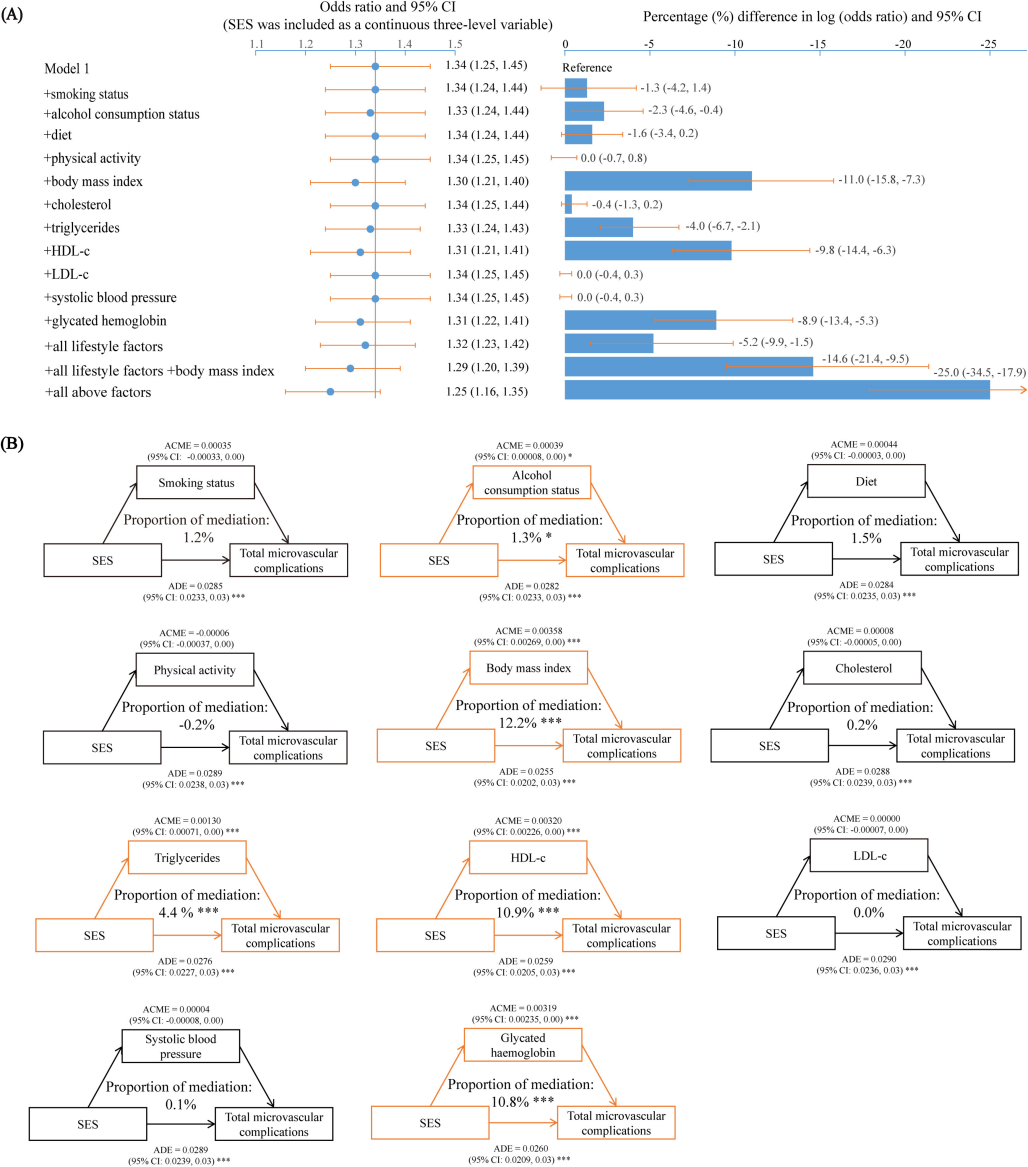
Association between SES, modifiable factors, and total microvascular complications. (A) Contribution of modifiable factors in explaining socioeconomic inequalities in total microvascular complications risk. Model 1: Adjusted for age, sex, ethnicity, blood glucose, diabetes duration, insulin use, medication for cholesterol, and medication for blood pressure. Percentage difference in log (odds ratio) = 100 × (*β*_SES+modifiable factor(s)_ − *β*_SES_)/(*β*_SES_), where *β* = log (odds ratio). (B) Mediation effect of modifiable factors on the association between SES and total microvascular complications risk. The mediation analysis was carried out by the “mediation” package in R, with 1000 simulations. ACME, average causal mediation effects; ADE, average direct effects; CI, confidence interval; HDL‐c, high density lipoprotein cholesterol; LDL‐c, low density lipoprotein cholesterol; SES, socioeconomic status.

Figure 2A illustrated the contributions of modifiable factors to the association between SES and total microvascular complications. In the base model, participants with lower SES had an odds ratio of 1.34 (95% CI: 1.25, 1.45) for total microvascular complications compared to those with higher SES. When alcohol consumption status, body mass index, triglycerides, high‐density lipoprotein cholesterol, and glycated hemoglobin were individually added to the model, the *β*_SES_ decreased by 2.3% (95% CI: 0.4%, 4.6%), 11.0% (95% CI: 7.3%, 15.8%), 4.0% (95% CI: 2.1%, 6.7%), 9.8% (95% CI: 6.3%, 14.4%), and 8.9% (95% CI: 5.3%, 13.4%), respectively. Incorporating all lifestyle factors resulted in a collective reduction of 5.2% (95% CI: 1.5%, 9.9%) in *β*_SES_. Adding all relevant modifiable factors led to a combined reduction in *β*_SES_ of 25.0% (95% CI: 17.9%, 34.5%). Contributions of modifiable factors to the association between SES and individual microvascular complications could be found in Table S4.

The association between SES and mediators, as well as the association between mediators and diabetic microvascular complications, could be found in Tables S5 and S6, respectively. Mediation analysis results indicated that alcohol consumption status, body mass index, triglycerides, high density lipoprotein cholesterol, and glycated hemoglobin mediated the association between SES and total microvascular complications, with mediation percentages of 1.3% (95% CI: 0.3%, 3.0%), 12.2% (95% CI: 8.4%, 17.0%), 4.4% (95% CI: 2.4%, 7.0%), 10.9% (95% CI: 7.1%, 16.0%), and 10.8% (95% CI: 7.4%, 16.0%), respectively (Figure 2B).

Body mass index, high density lipoprotein cholesterol, and glycated hemoglobin also mediated the association between SES and individual microvascular complications. The mediating proportion of body mass index in the association between SES and nephropathy, retinopathy, and neuropathy was 10.1% (95% CI: 6.9%, 14.0%), 16.5% (95% CI: 4.7%, 81.0%), and 14.1% (95% CI: 7.8%, 29.0%), respectively; The mediating proportion of high density lipoprotein cholesterol was 9.8% (95% CI: 6.6%, 15.0%), 16.0% (95% CI: 5.5%, 77.0%), and 7.2% (95% CI: 1.5%, 18.0%), respectively; and the mediating proportion of glycated hemoglobin was 5.2% (95% CI: 3.1%, 8.0%), 29.5% (95% CI: 14.8%, 143.0%), and 9.0% (95% CI: 4.6%, 18.0%), respectively (Table 2).

**TABLE 2:** Mediation effects of socioeconomic status on individual microvascular complications by modifiable factors.

Outcome	Mediator	ADE (95% CI)	ACME (95% CI)	Mediation proportion (%) (95% CI)
Nephropathy	Smoking status	0.0204 (0.0178, 0.02)***	0.00050 (0.00004, 0.00)*	2.4 (0.2, 5.0)*
	Alcohol consumption status	0.0206 (0.0182, 0.02)***	0.00021 (0.00002, 0.00)*	1.0 (0.1, 2.0)*
	Diet	0.0205 (0.0181, 0.02)***	0.00041 (0.00009, 0.00)**	1.8 (0.4, 4.0)**
	Physical activity	0.0209 (0.0184, 0.02)***	−0.00004 (−0.00026, 0.00)	−0.2 (−1.3, 1.0)
	Body mass index	0.0190 (0.0161, 0.02)***	0.00216 (0.00150, 0.00)***	10.1 (6.9, 14.0)***
	Cholesterol	0.0209 (0.0185, 0.02)***	0.00002 (−0.00008, 0.00)	0.1 (−0.4, 1.0)
	Triglycerides	0.0200 (0.0176, 0.02)***	0.00099 (0.00059, 0.00)***	4.6 (2.8, 7.0)***
	HDL‐c	0.0190 (0.0162, 0.02)***	0.00209 (0.00144, 0.00)***	9.8 (6.6, 15.0)***
	LDL‐c	0.0209 (0.0183, 0.02)***	−0.00000 (−0.00007, 0.00)	−0.0 (−0.3, 0.0)
	Systolic blood pressure	0.0209 (0.0184, 0.02)***	0.00000 (−0.00008, 0.00)	0.0 (−0.4, 0.0)
	Glycated hemoglobin	0.0199 (0.0172, 0.02)***	0.00110 (0.00068, 0.00)***	5.2 (3.1, 8.0)***
Retinopathy	Smoking status	0.0068 (0.0013, 0.01)*	−0.00011 (−0.00067, 0.00)	−1.4 (−17.1, 9.0)
	Alcohol consumption status	0.0065 (0.0009, 0.01)*	0.00018 (−0.00011, 0.00)	2.6 (−2.4, 16.0)
	Diet	0.0069 (0.0013, 0.01)*	0.00001 (−0.00036, 0.00)	0.2 (−7.4, 9.0)
	Physical activity	0.0069 (0.0014, 0.01)*	−0.00003 (−0.00024, 0.00)	−0.2 (−5.3, 3.0)
	Body mass index	0.0056 (−0.0002, 0.01)	0.00119 (0.00053, 0.00)***	16.5 (4.7, 81.0)*
	Cholesterol	0.0067 (0.0013, 0.01)*	0.00014 (−0.00001, 0.00)	1.9 (−0.3, 10.0)
	Triglycerides	0.0068 (0.0012, 0.01)*	0.00003 (−0.00043, 0.00)	0.5 (−9.4, 12.0)
	HDL‐c	0.0058 (0.0001, 0.01)*	0.00119 (0.00046, 0.00)**	16.0 (5.5, 77.0)**
	LDL‐c	0.0069 (0.0011, 0.01)*	0.00001 (−0.00010, 0.00)	0.1 (−2.0, 4.0)
	Systolic blood pressure	0.0068 (0.0017, 0.01)*	0.00012 (−0.00002, 0.00)	1.6 (−0.3, 8.0)
	Glycated hemoglobin	0.0048 (−0.0010, 0.01)	0.00212 (0.00152, 0.00)***	29.5 (14.8, 143.0)*
Neuropathy	Smoking status	0.0058 (0.0037, 0.01)***	0.00015 (−0.00015, 0.00)	2.5 (−2.4, 9.0)
	Alcohol consumption status	0.0060 (0.0039, 0.01)***	−0.00001 (−0.00011, 0.00)	−0.2 (−1.9, 2.0)
	Diet	0.0061 (0.0041, 0.01)***	−0.00016 (−0.00041, 0.00)	−2.6 (−8.2, 1.0)
	Physical activity	0.0060 (0.0042, 0.01)***	−0.00002 (−0.00012, 0.00)	−0.1 (−2.3, 1.0)
	Body mass index	0.0051 (0.0030, 0.01)***	0.00088 (0.00052, 0.00)***	14.1 (7.8, 29.0)***
	Cholesterol	0.0060 (0.0038, 0.01)***	−0.00002 (−0.00008, 0.00)	−0.2 (−1.5, 1.0)
	Triglycerides	0.0057 (0.0036, 0.01)***	0.00031 (0.00010, 0.00)*	5.0 (1.5, 11.0)*
	HDL‐c	0.0056 (0.0035, 0.01)***	0.00045 (0.00010, 0.00)*	7.2 (1.5, 18.0)*
	LDL‐c	0.0060 (0.0039, 0.01)***	−0.00000 (−0.00005, 0.00)	−0.0 (−0.9, 1.0)
	Systolic blood pressure	0.0060 (0.0041, 0.01)***	−0.00003 (−0.00011, 0.00)	−0.4 (−1.9, 0.0)
	Glycated hemoglobin	0.0055 (0.0034, 0.01)***	0.00056 (0.00030, 0.00)***	9.0 (4.6, 18.0)***

*Note:* Effect and proportion estimations were adjusted for age, sex, ethnicity, blood glucose, diabetes duration, insulin use, medication for cholesterol, and medication for blood pressure.

Abbreviations: ACME, average causal mediation effects; ADE, average direct effects; CI, confidence interval; HDL‐c, high density lipoprotein cholesterol; LDL‐c, low density lipoprotein cholesterol.

*p < 0.05, **p < 0.01, ***p < 0.001.

### 3.4 Subgroup and Sensitivity Analysis

In the subgroup of age, sex, and diabetes duration, no significant differences were found in the association between SES and total microvascular complications, as well as the association between SES and diabetic nephropathy. The association of SES with diabetic retinopathy and diabetic neuropathy was statistically significant in participants younger than 65 years, males, and participants with diabetes of less than 5 years (*p* < 0.05).

In sensitivity analyses, results were generally robust when excluding participants with outcomes diagnosed within 2 years after baseline and when the glomerular filtration rate was further adjusted. Similar results were observed in analyses that used the Townsend Deprivation Index to reflect the area‐level SES (Table 3).

**TABLE 3:** Subgroup analysis and sensitivity analysis regarding the risk of diabetic microvascular complications.

		Adjusted hazard ratios (95% confidence intervals)			
		Total microvascular complications	Nephropathy	Retinopathy	Neuropathy
Subgroup analysis					
Age (years)					
< 65	High SES	1.00 (Reference)	1.00 (Reference)	1.00 (Reference)	1.00 (Reference)
	Medium SES	1.37 (1.16, 1.62)***	1.58 (1.24, 2.02)***	1.45 (1.14, 1.85)**	1.06 (0.74, 1.53)
	Low SES	1.93 (1.64, 2.28)***	2.45 (1.93, 3.11)***	1.65 (1.29, 2.11)***	1.80 (1.27, 2.55)**
≥ 65	High SES	1.00 (Reference)	1.00 (Reference)	1.00 (Reference)	1.00 (Reference)
	Medium SES	1.17 (0.90, 1.52)	1.12 (0.80, 1.56)	1.01 (0.69, 1.47)	1.27 (0.66, 2.46)
	Low SES	1.56 (1.21, 2.01)**	1.80 (1.30, 2.47)***	1.05 (0.73, 1.53)	1.73 (0.91, 3.26)
Sex					
Male	High SES	1.00 (Reference)	1.00 (Reference)	1.00 (Reference)	1.00 (Reference)
	Medium SES	1.35 (1.14, 1.60)***	1.45 (1.14, 1.83)**	1.34 (1.06, 1.71)*	1.23 (0.85, 1.78)
	Low SES	1.82 (1.54, 2.15)***	2.20 (1.75, 2.76)***	1.42 (1.11, 1.82)**	2.07 (1.45, 2.96)***
Female	High SES	1.00 (Reference)	1.00 (Reference)	1.00 (Reference)	1.00 (Reference)
	Medium SES	1.17 (0.90, 1.52)	1.23 (0.85, 1.78)	1.18 (0.80, 1.73)	0.85 (0.46, 1.57)
	Low SES	1.57 (1.21, 2.02)**	1.88 (1.32, 2.67)***	1.32 (0.90, 1.92)	1.15 (0.64, 2.06)
Diabetes duration (years)					
< 5	High SES	1.00 (Reference)	1.00 (Reference)	1.00 (Reference)	1.00 (Reference)
	Medium SES	1.35 (1.15, 1.59)***	1.38 (1.10, 1.73)**	1.40 (1.11, 1.77)**	1.28 (0.88, 1.86)
	Low SES	1.81 (1.54, 2.12)***	2.21 (1.78, 2.75)***	1.42 (1.12, 1.80)**	1.98 (1.37, 2.86)***
≥ 5	High SES	1.00 (Reference)	1.00 (Reference)	1.00 (Reference)	1.00 (Reference)
	Medium SES	1.14 (0.84, 1.53)	1.40 (0.93, 2.11)	1.01 (0.65, 1.56)	0.82 (0.46, 1.46)
	Low SES	1.54 (1.16, 2.06)**	1.82 (1.22, 2.70)**	1.27 (0.84, 1.93)	1.31 (0.76, 2.25)
Sensitivity analysis					
Excluding participants with outcomes diagnosed within 2 years after baseline (*n* = 11 079)					
	High SES	1.00 (Reference)	1.00 (Reference)	1.00 (Reference)	1.00 (Reference)
	Medium SES	1.29 (1.11, 1.49)**	1.36 (1.11, 1.67)**	1.26 (1.02, 1.56)*	1.14 (0.82, 1.59)
	Low SES	1.72 (1.49, 1.99)***	2.09 (1.72, 2.54)***	1.33 (1.07, 1.65)**	1.71 (1.24, 2.36)**
Further adjusting for the glomerular filtration rate (*n* = 11 306)^a^					
	High SES	1.00 (Reference)	1.00 (Reference)	1.00 (Reference)	1.00 (Reference)
	Medium SES	1.30 (1.13, 1.49)***	1.37 (1.12, 1.67)***	1.30 (1.06, 1.60)*	1.14 (0.83, 1.56)
	Low SES	1.76 (1.53, 2.02)***	2.08 (1.71, 2.51)***	1.40 (1.14, 1.72)**	1.79 (1.32, 2.43)***
Using the Townsend deprivation index to reflect the area‐level SES (*n* = 11 288)					
	Tertile 1	1.00 (Reference)	1.00 (Reference)	1.00 (Reference)	1.00 (Reference)
	Tertile 2	1.05 (0.94, 1.17)	1.08 (0.94, 1.24)	1.03 (0.87, 1.22)	1.21 (0.92, 1.58)
	Tertile 3	1.28 (1.15, 1.42)***	1.30 (1.13, 1.49)***	1.21 (1.02, 1.43)*	2.05 (1.60, 2.64)***

*Note:* Hazard ratios were calculated by adjusting age, sex, ethnicity, blood glucose, diabetes duration, insulin use, medication for cholesterol, and medication for blood pressure.

Abbreviation: SES, socioeconomic status.

^a^ For total microvascular complications and diabetic nephropathy, hazard ratios were calculated by adjusting age, sex, ethnicity, blood glucose, diabetes duration, insulin use, medication for cholesterol, medication for blood pressure, and glomerular filtration rate. For diabetic retinopathy and diabetic neuropathy, hazard ratios were calculated by adjusting age, sex, ethnicity, blood glucose, diabetes duration, insulin use, medication for cholesterol, and medication for blood pressure.

*p < 0.05, **p < 0.01, ***p < 0.001.

## 4 Discussion

To our knowledge, we are the first to systematically evaluate the longitudinal association between SES and diabetic microvascular complications as well as to explore potential mediating effects. This study yielded three key findings: first, compared to individuals with high SES, those with medium and low SES had a 30% and 75% increased risk of total microvascular complications; second, this positive association was also observed for individual microvascular complications; third, body mass index and other obesity‐related indicators, as well as glycated hemoglobin may be significant mediators of the association between SES and microvascular complications, with lifestyle factors having relatively smaller mediating proportions.

This study found a significant association between SES and diabetic microvascular complications. No previous research has utilized latent class analysis to define SES and explored this association, but alternative methods were employed in prior studies, with most results in line with ours. Among them, there was a body of research on diabetic nephropathy. Some studies used education, occupation, and income as components, and employed weighted scoring methods to represent SES [10], while others assessed economic hardship based on difficulties paying bills, food insecurity, and cost‐related medication non‐adherence [26], all of which found a positive association between low SES and diabetic nephropathy. Studies representing SES through educational attainment or evaluation of precarity and inequalities in health examination centers (EPICES) score also found an association between SES and diabetic retinopathy [9, 27]. There was relatively less literature on the association between SES and diabetic neuropathy. In a cross‐sectional study involving 135 diabetic patients, those with higher levels of poverty were found to have a greater risk of neuropathy compared to those with lower levels of poverty [9]. However, there were also a few studies that diverged from our findings. In the study by Chaturvedi et al. [13], there was little difference in the occurrence of microvascular complications among female patients with different education levels. The patients included in this study were predominantly insulin‐dependent, and the assessment of SES lacked occupational indicators and income, which may explain the inconsistencies with our study results.

Mediation analysis results suggested that obesity‐related indicators and glycated hemoglobin may be more significant mediators than lifestyle factors of the association between SES and diabetic microvascular complications. Previous studies have shown associations between glycated hemoglobin levels and SES as well as microvascular complications [28, 29]. The association between obesity and SES was also confirmed by some studies, with higher obesity rates observed among individuals of lower SES, a phenomenon particularly pronounced in developed countries [30], while the risk of microvascular complications was significantly higher among obese individuals [31, 32]. Additionally, our study revealed that lifestyle factors had some impact on the association between SES and microvascular complications, which was consistent with current evidence, although to a lesser extent. For instance, one study suggested that the distribution of detrimental health behaviors may explain the high mortality rates associated with low SES in the United States, highlighting the importance of social inequality in unhealthy behaviors [33], and Geng et al. [23] found a significant reduction in the risk of microvascular complications associated with adherence to healthy lifestyle behaviors.

Main strengths of this study included the large sample size, the long follow‐up period, and the comprehensive definition of individual‐level SES. In addition, we explored for the first time the potential mediators between SES and diabetic microvascular complications. We acknowledged that our study had some limitations. First, the information on SES and lifestyle factors was collected by touchscreen questionnaires, and recall bias was inevitable. Additionally, only baseline survey data were used to define each participant's SES and lifestyle. While this approach helped establish the temporal sequence between the exposure and diabetic microvascular complications [4], it did not fully account for potential changes in SES and lifestyle over the 12.2 years of follow‐up. Second, although we have made efforts to adjust for potential confounders, the association between SES and diabetic microvascular complications may also be affected by residual confounding factors, such as healthcare access quality [34, 35], medication adherence [36], and social support networks [37, 38]. Third, diabetic microvascular complications were identified through hospital admission records and death registries, which may result in underreporting of these complications [23, 39, 40]. Specifically, hospital diagnoses typically have high specificity but may exhibit low sensitivity for the complications studied [41], potentially leading to misclassification. This misclassification might result in an underestimation of the associations between SES and the risk of diabetic microvascular complications. Lastly, UK Biobank is acknowledged to have a low response rate (5.5%), with participants being more affluent and more likely to be from a white ethnic background than the UK population as a whole [42, 43]. Therefore, our data may not fully represent the broader UK population or other ethnic groups.

In summary, our study indicated that lower SES may be associated with a higher risk of developing microvascular complications among T2D patients, and that obesity‐related indicators and glycated hemoglobin may play significant mediating roles. SES is relatively difficult to change, and efforts are suggested to be made through other approaches such as increasing financial support for diabetic patients of lower SES and enhancing weight and blood glucose management to prevent or reduce the occurrence of diabetic microvascular complications.

## Author Contributions

Y.L. and Q.Y. have contributed equally to this work and share first authorship. Conceptualization: S.X., Y.L., and Q.Y. Methodology: S.X., Y.L., and Q.Y. Formal analysis: Y.L. and Q.Y. Writing – original draft: S.X., Y.L., and Q.Y. Writing – review and editing: S.X., Y.L., Q.Y., M.F., L.W., J.Z., B.C., and J.W. All authors read and approved the final paper draft.

## Conflicts of Interest

The authors declare no conflicts of interest.

## Supporting information


Data S1.

## Data Availability

Data set of this study came from the UK Biobank Database (https://www.ukbiobank.ac.uk), and access to the data can be obtained by contacting the UK Biobank Data Service. Ethical clearance for UK Biobank data was obtained from the North West Multi‐Centre Research Ethics Committee (REC reference: 21/NW/0157). This study was conducted based on a data analysis application (application number: 92014).
