# Supplementary material for: Socioeconomic Status, Modifiable Factors, and Risk of Microvascular Complications in Individuals With Type 2 Diabetes: A Cohort Study From the UK Biobank
Source: J Diabetes. 2025 Apr 2;17(4):e70079. doi: 10.1111/1753-0407.70079 (PMC11962517; doi:10.1111/1753-0407.70079)

**List of supplementary material**

**Table S1** Definitions of socioeconomic status, lifestyle factors and diabetic microvascular complications from UK Biobank

**Table S2** Latent class analysis index in the cluster selection

**Table S3** Mean posterior probabilities, prevalence of latent classes, and item-response probabilities in model

**Table S4** Contribution of modifiable factors in explaining socioeconomic inequalities in individual microvascular complications risk

**Table S5** Association between socioeconomic status and modifiable factors

**Table S6** Association between modifiable factors and diabetic microvascular complications

**Figure S1** Study flow chart

**Table S1** Definitions of socioeconomic status, lifestyle factors and diabetic microvascular complications from UK Biobank

| **Variables** | **Sub-variables (code)** | **Definition** |
| --- | --- | --- |
| Socioeconomic status ^1^ | Average total household income before tax (£) (738) | Grouped into 5 levels:  Less than 18,000;  18,000 to 30,999;  31,000 to 51,999;  52,000 to 100,000;  Greater than 100,000 |
|  | Education (6138) | Grouped into 7 levels:  College or University degree;  A levels/AS levels or equivalent;  O levels/GCSEs or equivalent;  CSEs or equivalent;  NVQ or HND or HNC, or equivalent;  Other professional qualifications;  None of the above (equivalent to less than high school diploma) |
|  | Employment status (6142) | Employed status was defined as meeting one of the following:  In paid employment or self-employed;  Retired;  Doing unpaid or voluntary work;  Full or part-time student |
| Lifestyle factors ^2,3^ | Smoking status  (20116, 2897) | No current smoking was defined as meeting one of the following:  Never smoking;  Former smoking but had quit for more than 30 years |
|  | Alcohol consumption status  (20117) | No alcohol consumption was defined as:  Never alcohol drinking |
|  | Diet  (1289, 1299, 1309, 1319, 1329, 1339, 1349, 1369, 1379, 1389, 1438, 1448, 1458, 1468) | Healthy diet was defined as meeting at least 4 of the following:  Fruits >= 4 servings/day;  Vegetables >= 4 servings/day;  Fish >= 2 times/week;  Processed meats < = 1 times/week;  Unprocessed red meats < = 1.5 times/week;  Whole grains > = 3 servings/day  (Amount of per serving was defined as: fresh fruit: 1 piece; dried fruit: 5 pieces; cooked/raw vegetables: 3 heaped tablespoons; bran/oat/muesli cereal: 1 bowl/day; wholemeal/wholegrain bread: 1 slice/day) |
|  | Physical activity  (894, 914, 884, 904) | Regular physical activity was defined as meeting at least one of the following:  >= 150 minutes/week of moderate activity;  >= 75 minutes/week of vigorous activity;  (moderate activity + 2*vigorous activity) >= 150 minutes/week |
| Microvascular complications ^4^ | Diabetic nephropathy (41270) | ICD 10 codes: E11.2, E14.2, N08.3, N18.0, N18.1, N18.2, N18.3, N18.4, N18.5, N18.8, N18.9 |
|  | Diabetic retinopathy (41270) | ICD 10 codes: E11.3, E14.3, H28.0, H36.0 |
|  | Diabetic neuropathy (41270) | ICD 10 codes: E11.4, E14.4, G59.0, G62.9, G63.2, G99.0 |

ICD 10, International Classification of Diseases

**Reference**

1 Zhang YB, Chen C, Pan XF, et al. Associations of healthy lifestyle and socioeconomic status with mortality and incident cardiovascular disease: two prospective cohort studies. Bmj. Apr 14 2021;373:n604.

2 Ye X, Wang Y, Zou Y, et al. Associations of socioeconomic status with infectious diseases mediated by lifestyle, environmental pollution and chronic comorbidities: a comprehensive evaluation based on UK Biobank. Infect Dis Poverty. Jan 30 2023;12(1):5.

3 Han H, Cao Y, Feng C, et al. Association of a Healthy Lifestyle With All-Cause and Cause-Specific Mortality Among Individuals With Type 2 Diabetes: A Prospective Study in UK Biobank. Diabetes Care. Feb 1 2022;45(2):319-329.

4 Geng T, Zhu K, Lu Q, et al. Healthy lifestyle behaviors, mediating biomarkers, and risk of microvascular complications among individuals with type 2 diabetes: A cohort study. PLoS Med. Jan 2023;20(1):e1004135.

**Table S2** Latent class analysis index in the cluster selection

| **Cluster** | **X^2^** | **AIC** | **BIC** | **G^2^** |
| --- | --- | --- | --- | --- |
| 2-cluster | 424.42 | 77017.75 | 77186.42 | 433.12 |
| 3-cluster | 99.73 | 76701.73 | 76958.40 | 93.10 |
| 4-cluster | 49.42 | 76681.04 | 77025.70 | 48.41 |
| 5-cluster | 29.76 | 76687.86 | 77120.52 | 31.22 |

X^2^, Akaike information criterion (AIC), Bayesian information criterion (BIC), and likelihood ratio statistic G^2^ were used for the model selection.

As the X^2^ and G^2^ decreases, the BIC in the 3-cluster model reached bottom and then increases. Thus, we chose the 3-cluster model in the present analysis.

**Table S3** Mean posterior probabilities, prevalence of latent classes, and item-response probabilities in model

| **Item** | **Latent class 1** | **Latent class 2** | **Latent class 3** |
| --- | --- | --- | --- |
| Mean posterior probability | 0.21 | 0.39 | 0.41 |
| Prevalence | 0.17 | 0.40 | 0.42 |
| Greater than ₤100000 | 0.16 | 0.01 | 0.00 |
| ₤52000-100000 | 0.48 | 0.01 | 0.12 |
| ₤31000-51999 | 0.35 | 0.04 | 0.35 |
| ₤18000-30999 | 0.01 | 0.18 | 0.51 |
| Less than ₤18000 | 0.00 | 0.76 | 0.03 |
| College or university degree | 0.70 | 0.14 | 0.28 |
| A/AS levels or equivalent | 0.15 | 0.07 | 0.12 |
| O/GCSEs level or equivalent | 0.07 | 0.20 | 0.26 |
| CSEs or equivalent | 0.01 | 0.06 | 0.06 |
| NVQ/HND/HNC or equivalent | 0.04 | 0.09 | 0.11 |
| Other professional qualifications | 0.04 | 0.06 | 0.08 |
| None of the above | 0.00 | 0.38 | 0.10 |
| Employed | 0.97 | 0.81 | 0.99 |
| Unemployed | 0.03 | 0.19 | 0.01 |
| Socioeconomic status group | High | Low | Medium |

Total household income before tax, education level, and employment status were used to generate an overall individual socioeconomic status parameter. The mean posterior probability reflected the uncertainty of posterior classification. Prevalence indicated the prevalence of each latent class. Item-response probability was a posterior probability and was used for defining latent classes.

**Table S4** Contribution of modifiable factors in explaining socioeconomic inequalities in individual microvascular complications risk

|  | **Odds ratio (95% CI)** † | **% difference (95% CI)** ‡ |
| --- | --- | --- |
| **Nephropathy** | | |
| **Model 1** | 1.49 (1.36, 1.63) *** | Reference |
| Model 1 + smoking status | 1.47 (1.34, 1.61) *** | **-2.6 (-5.3, -0.2)** |
| Model 1 + alcohol consumption status | 1.48 (1.35, 1.62) *** | **-1.6 (-3.7, -0.1)** |
| Model 1 + diet | 1.48 (1.35, 1.62) *** | **-1.9 (-3.7, -0.5)** |
| Model 1 + physical activity | 1.49 (1.36, 1.63) *** | -0.1 (-0.9, 0.7) |
| Model 1 + body mass index | 1.44 (1.31, 1.58) *** | **-9.0 (-12.9, -5.9)** |
| Model 1 + cholesterol | 1.49 (1.36, 1.63) *** | -0.2 (-1.1, 0.7) |
| Model 1 + triglycerides | 1.47 (1.34, 1.61) *** | **-3.5 (-5.9, -1.9)** |
| Model 1 + high density lipoprotein cholesterol | 1.44 (1.31, 1.58) *** | **-8.5 (-12.7, -5.3)** |
| Model 1 + low density lipoprotein cholesterol | 1.49 (1.36, 1.63) *** | 0.2 (-0.4, 0.8) |
| Model 1 + systolic blood pressure | 1.49 (1.36, 1.63) *** | 0.0 (-0.2, 0.3) |
| Model 1 + glycated haemoglobin | 1.47 (1.34, 1.61) *** | **-3.9 (-6.2, -2.2)** |
| **Model 2:** model 1 + all lifestyle factors | 1.45 (1.32, 1.59) *** | **-6.3 (-10.7, -2.6)** |
| **Model 3:** model 1 + all lifestyle factors + body mass index | 1.41 (1.29, 1.55) *** | **-13.7 (-19.8, -9.0)** |
| **Model 4:** all above factors | 1.37 (1.25, 1.51) *** | **-20.0 (-26.9, -14.3)** |
| **Retinopathy** | | |
| **Model 1** | 1.13 (1.02, 1.25) * | Reference |
| Model 1 + smoking status | 1.13 (1.02, 1.26) * | 2.2 (-12.2, 18.7) |
| Model 1 + alcohol consumption status | 1.13 (1.02, 1.25) * | -6.7 (-25.1, 2.4) |
| Model 1 + diet | 1.13 (1.02, 1.25) * | -0.6 (-10.2, 9.9) |
| Model 1 + physical activity | 1.13 (1.02, 1.25) * | -0.3 (-2.6, 2.4) |
| Model 1 + body mass index | 1.11 (1.00, 1.23) * | **-18.5 (-62.5, -4.8)** |
| Model 1 + cholesterol | 1.13 (1.02, 1.25) * | -3.0 (-9.9, 0.4) |
| Model 1 + triglycerides | 1.13 (1.02, 1.25) * | 0.8 (-9.0, 11.9) |
| Model 1 + high density lipoprotein cholesterol | 1.11 (1.00, 1.23) * | **-16.3 (-51.7, -5.0)** |
| Model 1 + low density lipoprotein cholesterol | 1.13 (1.02, 1.25) * | -0.3 (-4.2, 2.6) |
| Model 1 + systolic blood pressure | 1.13 (1.02, 1.25) * | -1.1 (-6.7, 2.2) |
| Model 1 + glycated haemoglobin | 1.10 (0.99, 1.21) | **-33.6 (-95.4, -11.1)** |
| **Model 2:** model 1 + all lifestyle factors | 1.13 (1.02, 1.25) * | -1.5 (-22.9, 12.6) |
| **Model 3:** model 1 + all lifestyle factors + body mass index | 1.11 (1.00, 1.23) * | **-19.0 (-67.4, -1.4)** |
| **Model 4:** all above factors | 1.07 (0.97, 1.19) | **-50.9 (-159.7, -17.3)** |
| **Neuropathy** | | |
| **Model 1** | 1.36 (1.17, 1.58) *** | Reference |
| Model 1 + smoking status | 1.35 (1.16, 1.57) *** | -2.8 (-9.8, 3.1) |
| Model 1 + alcohol consumption status | 1.36 (1.17, 1.58) *** | 0.2 (-4.3, 5.1) |
| Model 1 + diet | 1.37 (1.18, 1.59) *** | 3.4 (-0.3, 9.4) |
| Model 1 + physical activity | 1.36 (1.17, 1.58) *** | -0.2 (-1.5, 0.9) |
| Model 1 + body mass index | 1.30 (1.12, 1.51) ** | **-15.6 (-32.1, -7.3)** |
| Model 1 + cholesterol | 1.36 (1.17, 1.58) *** | 0.2 (-0.7, 1.4) |
| Model 1 + triglycerides | 1.34 (1.15, 1.56) *** | **-5.3 (-12.3, -1.5)** |
| Model 1 + high density lipoprotein cholesterol | 1.33 (1.15, 1.55) *** | **-6.7 (-17.0, -1.0)** |
| Model 1 + low density lipoprotein cholesterol | 1.36 (1.17, 1.58) *** | 0.0 (-0.9, 0.8) |
| Model 1 + systolic blood pressure | 1.36 (1.17, 1.58) *** | 0.0 (-1.0, 1.0) |
| Model 1 + glycated haemoglobin | 1.32 (1.14, 1.54) *** | **-9.2 (-19.1, -3.7)** |
| **Model 2:** model 1 + all lifestyle factors | 1.36 (1.17, 1.58) *** | 0.6 (-7.7, 9.5) |
| **Model 3:** model 1 + all lifestyle factors + body mass index | 1.31 (1.12, 1.52) *** | **-13.7 (-31.2, -3.8)** |
| **Model 4:** all above factors | 1.28 (1.10, 1.49) ** | **-21.5 (-44.7, -8.7)** |

Model 1 was adjusted for age, sex, ethnicity, blood glucose, diabetes duration, insulin use, medication for cholesterol, and medication for blood pressure

† Socioeconomic status was included in the regression model as a continuous three-level variable. Odds ratio was for the lowest versus highest socioeconomic status (reference).

‡ Percentage difference in log (odds ratio) = 100 * (*β* _SES + modifiable factor(s)_ - *β* _SES_) / (*β* _SES_), where *β* = log (odds ratio). Percentage difference and corresponding 95% CI were calculated by using a bootstrap method with 1,000 re-samplings

CI, confidence interval

**P* < 0.05, ***P* < 0.01, ****P* < 0.001

**Table S5** Association between socioeconomic status and modifiable factors

| **Modifiable factors (dependent variable)** | **Odds ratio (95% confidence interval)** † | ***P*-value** |
| --- | --- | --- |
| No current smoking | 0.72 (0.68, 0.76) | <0.001 |
| No alcohol consumption | 1.65 (1.47, 1.86) | <0.001 |
| Healthy diet | 0.82 (0.78, 0.87) | <0.001 |
| Regular physical activity | 1.02 (0.96, 1.08) | 0.497 |
| Body mass index | 2.30 (2.01, 2.63) | <0.001 |
| Cholesterol | 0.98 (0.95, 1.00) | 0.065 |
| Triglycerides | 1.16 (1.12, 1.19) | <0.001 |
| High density lipoprotein cholesterol | 0.95 (0.94, 0.96) | <0.001 |
| Low density lipoprotein cholesterol | 1.00 (0.98, 1.02) | 0.822 |
| Systolic blood pressure | 1.47 (0.95, 2.28) | 0.081 |
| Glycated haemoglobin | 4.37 (3.25, 5.87) | <0.001 |

Socioeconomic status was included in the regression model as a continuous three-level variable.

† Binary logistic or multiple linear regression models with the modifiable factors as the dependent variable and socioeconomic status as the independent variable. Adjusted for age, sex, ethnicity, blood glucose, diabetes duration, insulin use, medication for cholesterol, and medication for blood pressure

**Table S6** Association between modifiable factors and diabetic microvascular complications

| **Modifiable factors**  **(independent variable)** | **Odds ratio (95% confidence interval)** † | | | |
| --- | --- | --- | --- | --- |
|  | **Total microvascular complications** | **Nephropathy** | **Retinopathy** | **Neuropathy** |
| No current smoking | 0.95 (0.85, 1.05) | 0.87 (0.76, 0.98) * | 1.03 (0.89, 1.20) | 0.90 (0.73, 1.11) |
| No alcohol consumption | 1.29 (1.06, 1.58) * | 1.30 (1.03, 1.65) * | 1.20 (0.92, 1.57) | 0.97 (0.62, 1.52) |
| Healthy diet | 0.91 (0.82, 1.00) | 0.85 (0.75, 0.96) * | 0.99 (0.86, 1.15) | 1.20 (0.97, 1.48) |
| Regular physical activity | 0.86 (0.76, 0.96) ** | 0.83 (0.72, 0.96) ** | 0.89 (0.76, 1.05) | 0.87 (0.69, 1.10) |
| Body mass index | 1.04 (1.04, 1.05) *** | 1.05 (1.04, 1.06) *** | 1.03 (1.01, 1.04) *** | 1.05 (1.04, 1.07) *** |
| Cholesterol | 0.97 (0.92, 1.02) | 0.99 (0.93, 1.05) | 0.91 (0.84, 0.98) ** | 1.03 (0.93, 1.14) |
| Triglycerides | 1.10 (1.05, 1.14) *** | 1.14 (1.09, 1.19) *** | 1.00 (0.95, 1.06) | 1.11 (1.04, 1.19) ** |
| High density lipoprotein cholesterol | 0.53 (0.44, 0.63) *** | 0.46 (0.37, 0.58) *** | 0.66 (0.52, 0.85) ** | 0.65 (0.45, 0.93) * |
| Low density lipoprotein cholesterol | 1.00 (0.93, 1.07) | 1.03 (0.94, 1.12) | 0.91 (0.82, 1.00) | 1.05 (0.91, 1.21) |
| Systolic blood pressure | 1.00 (1.00, 1.00) | 1.00 (1.00, 1.00) | 1.01 (1.00, 1.01) ** | 1.00 (0.99, 1.00) |
| Glycated haemoglobin | 1.02 (1.02, 1.03) *** | 1.01 (1.01, 1.02) *** | 1.03 (1.02, 1.03) *** | 1.02 (1.01, 1.03) *** |

Binary logistic regression models with diabetic microvascular complications as the dependent variable

† Adjusted for age, sex, ethnicity, blood glucose, diabetes duration, insulin use, medication for cholesterol, medication for blood pressure, and socioeconomic status

**P* < 0.05, ***P* < 0.01, ****P* < 0.001

**Figure S1** Study flow chart


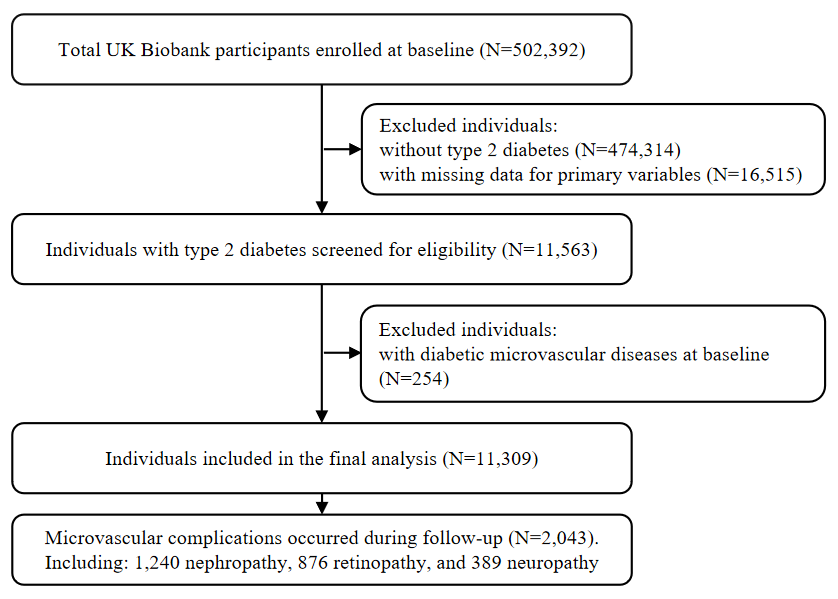

Supplement: Supplementary file 1 — Data S1. [file JDB-17-e70079-s001.docx]
